# Supplementary material for: Effects of Pitavastatin, Atorvastatin, and Rosuvastatin on the Risk of New-Onset Diabetes Mellitus: A Single-Center Cohort Study
Source: Biomedicines. 2020 Nov 13;8(11):499. doi: 10.3390/biomedicines8110499 (PMC7696728; doi:10.3390/biomedicines8110499)
Supplement: Supplementary file 1 [file biomedicines-08-00499-s001.pdf]

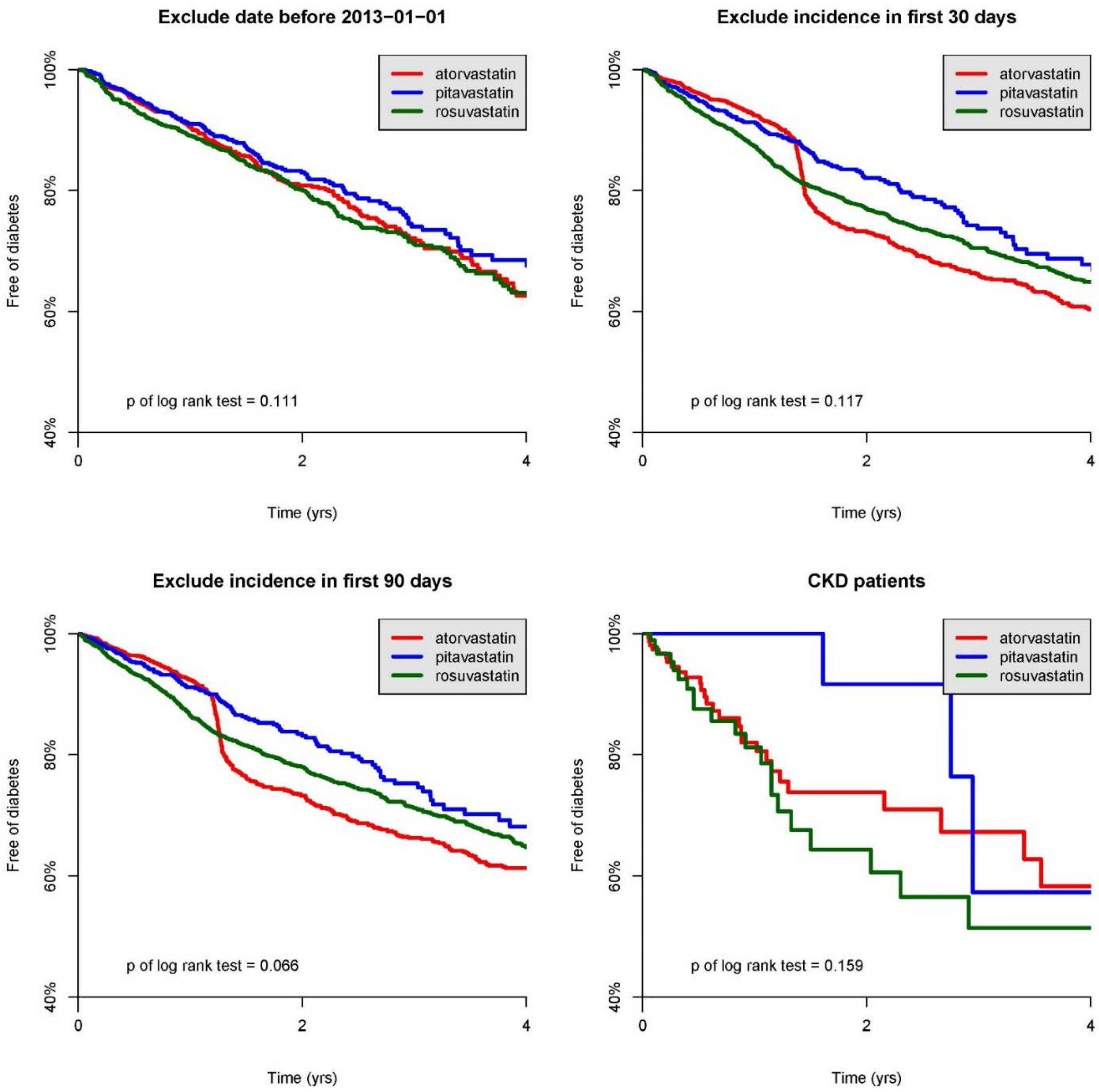

Supplementary Figure 1. Sensitivity test with Kaplan–Meier plot of three statin groups in specific circumstances for new-onset diabetes mellitus (NODM)-free probabilities curve. CKD: chronic kidney disease.

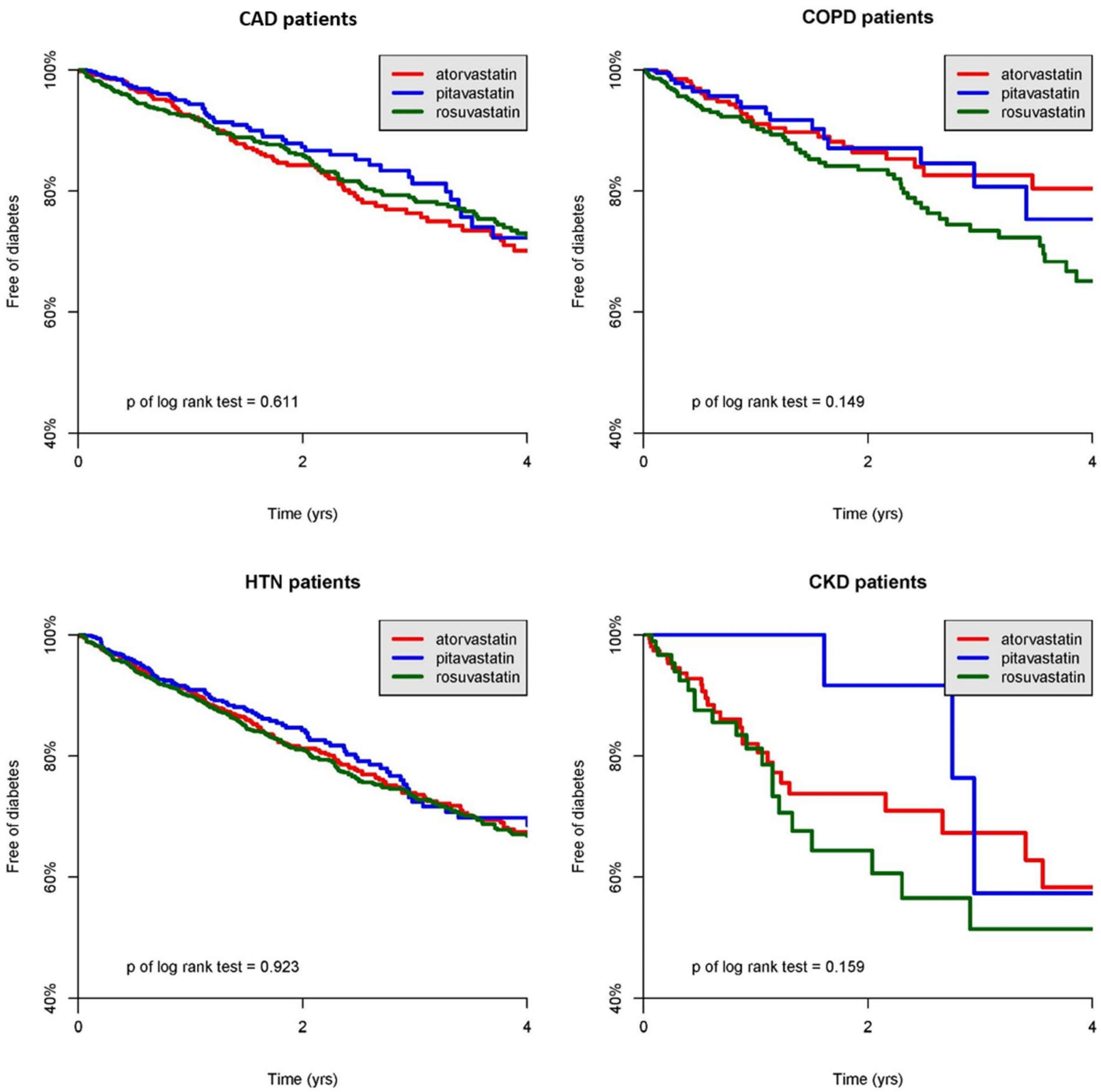

Supplementary Figure 2. Sensitivity test with Kaplan–Meier plot of three statin groups in specific circumstances for new-onset diabetes mellitus (NODM)-free probabilities curve. CAD: coronary artery disease; CKD: chronic kidney disease; COPD: chronic obstructive pulmonary disease; HTN: hypertension.

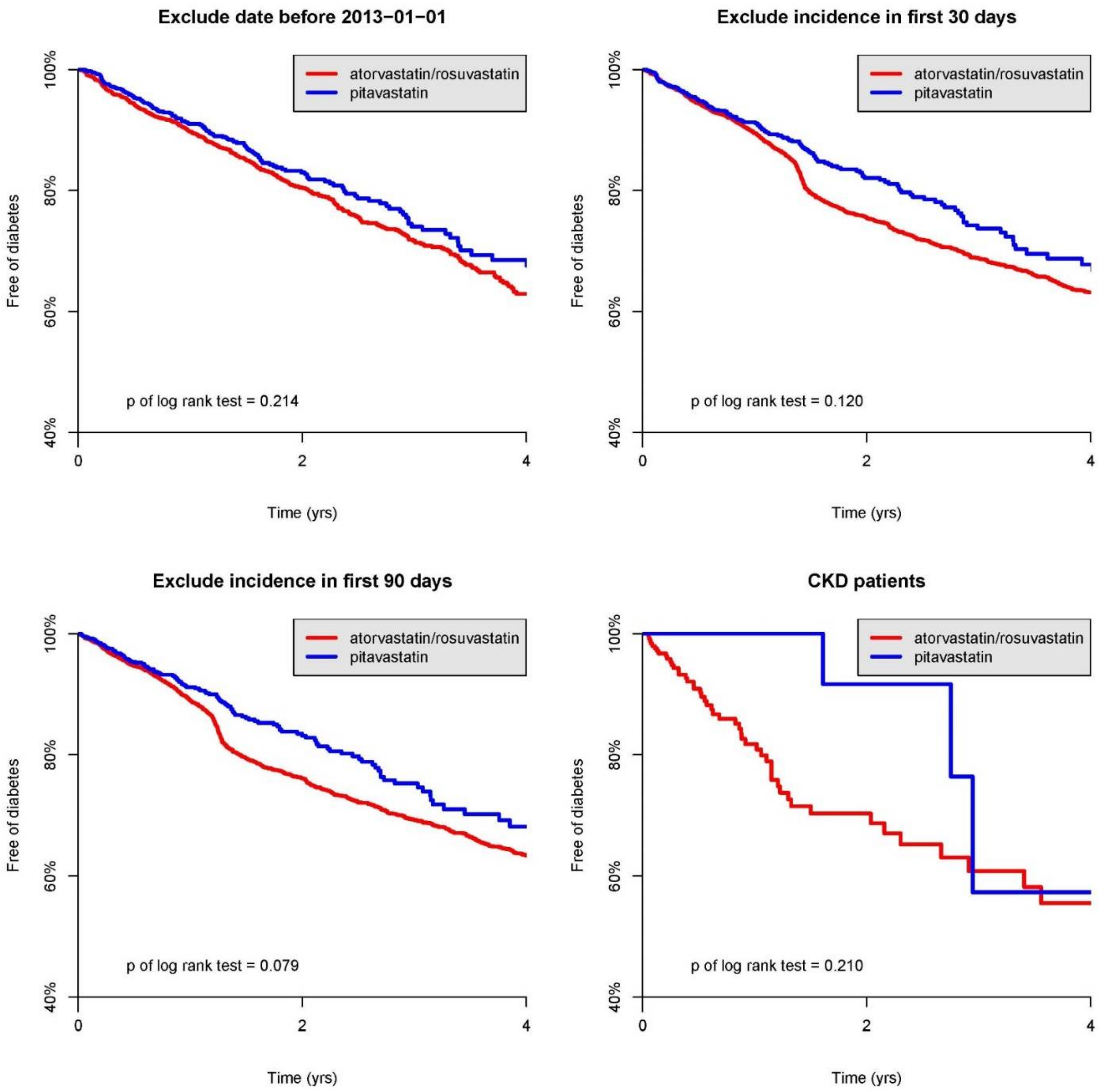

Supplementary Figure 3. Sensitivity test with Kaplan–Meier plot of pitavastatin and combination of atorvastatin and rosuvastatin in specific circumstances for new-onset diabetes mellitus (NODM)-free probabilities curve. CKD: chronic kidney disease.

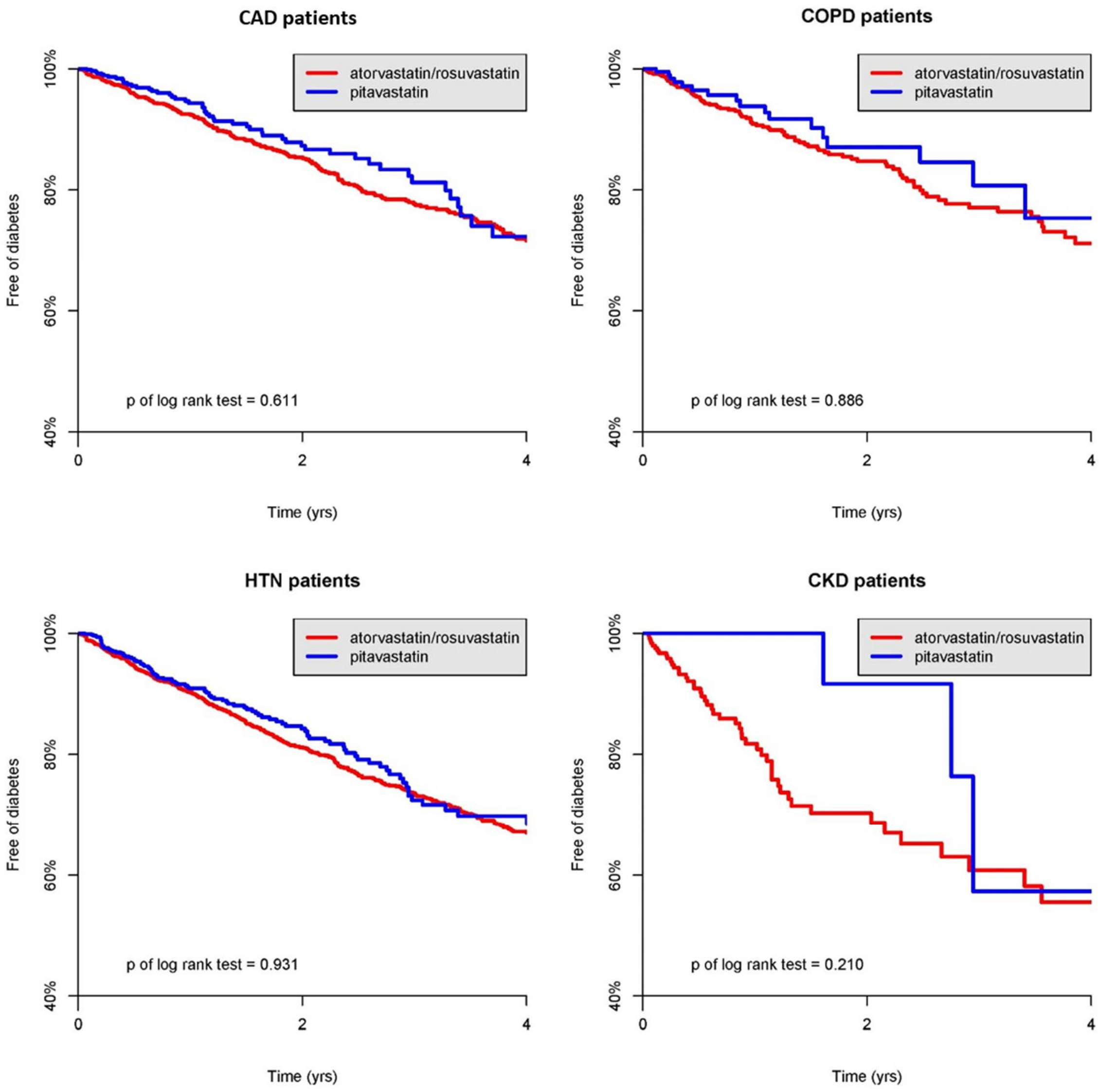

Supplementary Figure 4. Sensitivity test with Kaplan–Meier plot of pitavastatin and combination of atorvastatin and rosuvastatin in specific circumstances for new-onset diabetes mellitus (NODM)-free probabilities curve. CAD: coronary artery disease; CKD: chronic kidney disease; COPD: chronic obstructive pulmonary disease; HTN: hypertension.

Supplementary Table 1. The change in LDL levels of three statin groups in the follow-up periods.

Variables	Pitavastatin	Atorvastatin	Rosuvastatin	p-value
LDL (mg/dL)				
Baseline	131.52±28.51	122.94±37.57	141.30±45.86	<0.001
Six months	93.18±24.26	92.49±26.84	94.67±30.35	0.121
Last	97.97±28.08	97.85±32.72	102.39±37.88	<0.001
Reduction (%)	23%±25%	16%±35%	22%±33%	<0.001

LDL: low-density lipoprotein; Baseline indicates the baseline LDL levels; Six months indicates the LDL levels after six months of statin therapy; Last indicates the last LDL levels in the follow-up periods; Reduction indicates the percentage of reduction comparing the last LDL levels to the baseline ones.

Supplementary Table 2. HR in NODM of three statin groups who received LDL, cholesterol, triglyceride tests.

Independent variables	Crude-HR (95% CI)	p-value	Adj-HR (95% CI)#	p-value
Comparison 1		0.038		0.116
Pitavastatin	1.00		1.00	
Atorvastatin	1.21 (1.02 - 1.44)	0.032	1.09 (0.88 - 1.34)	0.437
Rosuvastatin	1.24 (1.05 - 1.47)	0.011	1.22 (1.00 - 1.50)	0.053
Comparison 2				
Pitavastatin	1.00		1.00	
Atorvastatin/ Rosuvastatin	1.23 (1.05 - 1.45)	0.012	1.16 (0.96 - 1.39)	0.131

#All results of Adj-HR were adjusted by years, gender, age, coronary artery disease, hypertension, chronic obstructive pulmonary disease, chronic kidney disease, cancer, ischemic stroke, hemorrhagic stroke, heart failure, LDL, cholesterol, triglyceride. Only patients who received LDL, cholesterol, triglyceride tests were included in the multivariate analysis (N = 5175). Adj-HR: adjusted hazard ratio; CI: confidence interval; HR: hazard ratio; LDL: low-density lipoprotein; N: number of patients NODM: new-onset diabetes mellitus; years: years before and after the release of pitavastatin.
